# Supplementary material for: Proneurogenic Actions of FSH During Directed Differentiation of Neural Stem and Progenitor Cells from Ovarian Cortical Cells Towards the Dopaminergic Pathway
Source: Biomedicines. 2025 Jun 26;13(7):1560. doi: 10.3390/biomedicines13071560 (PMC12292564; doi:10.3390/biomedicines13071560)
Supplement: Supplementary file 1 [file biomedicines-13-01560-s001.zip › biomedicines-3635010-supplementary.pdf]

Figure S1: Individual and combined immunolocalization of NeuN, TH, and DAPI in neurons derived from OCC-NSPC after directed differentiation to the DA pathway.

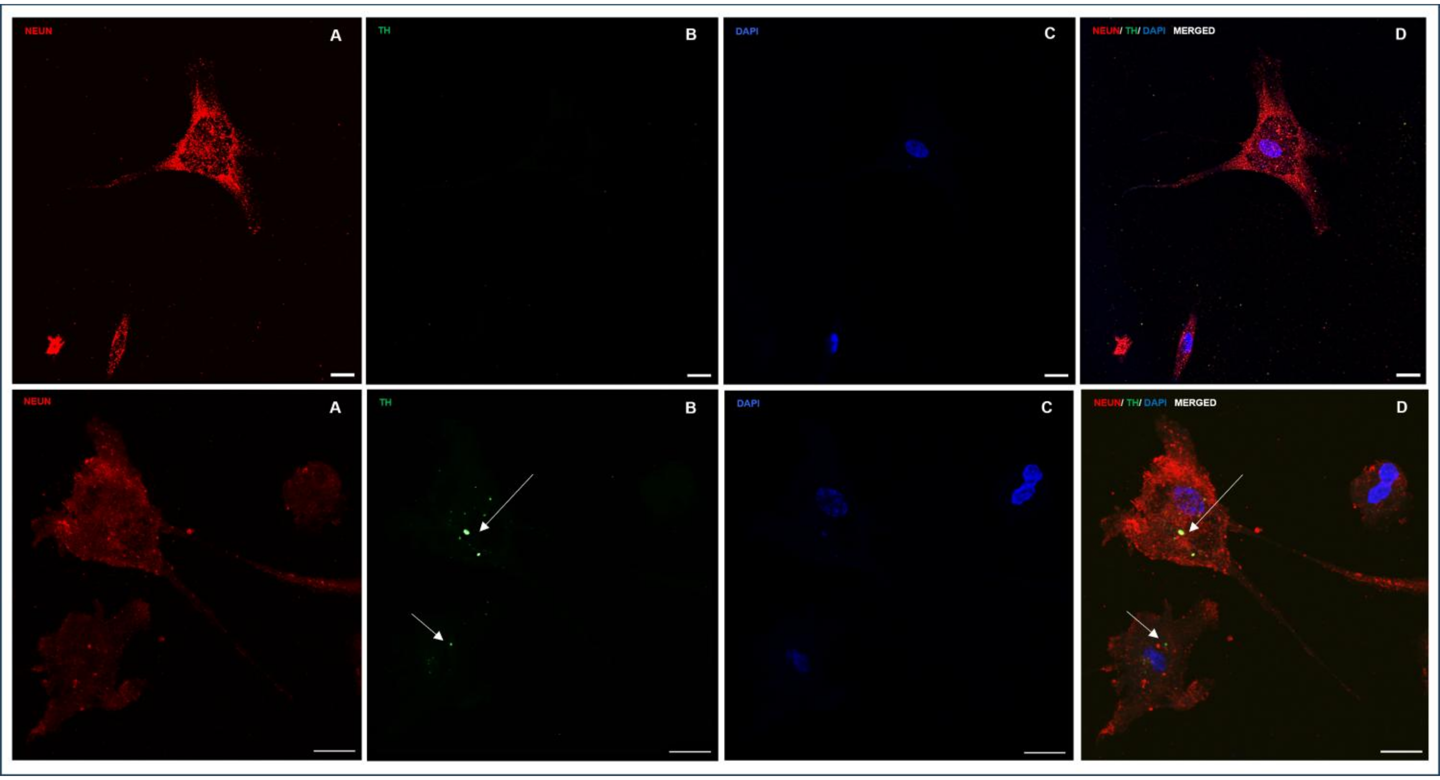

Figure S1.- Microphotographs showing immunolocalization of NeuN (A; red staining), TH (B; green staining), DAPI (C; blue staining, nuclei), and merged immunolocalization of NeuN, TH and DAPI (D) in neurons growing in culture after thirty days of directed differentiation of OCC-NSPCs to the DA pathway. Upper images (upper A-D), show a neuron with positive immunolocalization of NeuN (red; A, D), and absence of immunolocalization of TH (green). Lower images (lower A-D), show a neuron and a neuron precursor with positive immunolocalization of NeuN (red; A, D) and TH (B, D; white arrows). (HC, PL APO CS2, 63X/1.40 magnification, oil). Nuclei were counterstained with DAPI. This figure supplements Figure 6 of the manuscript.

Figure S2: Negative control, individual and combined immunolocalization of GFAP, NeuN, TH, and DAPI in astrocytes and DA neuron precursor after directed differentiation of OCC-NSPC to the DA pathway.

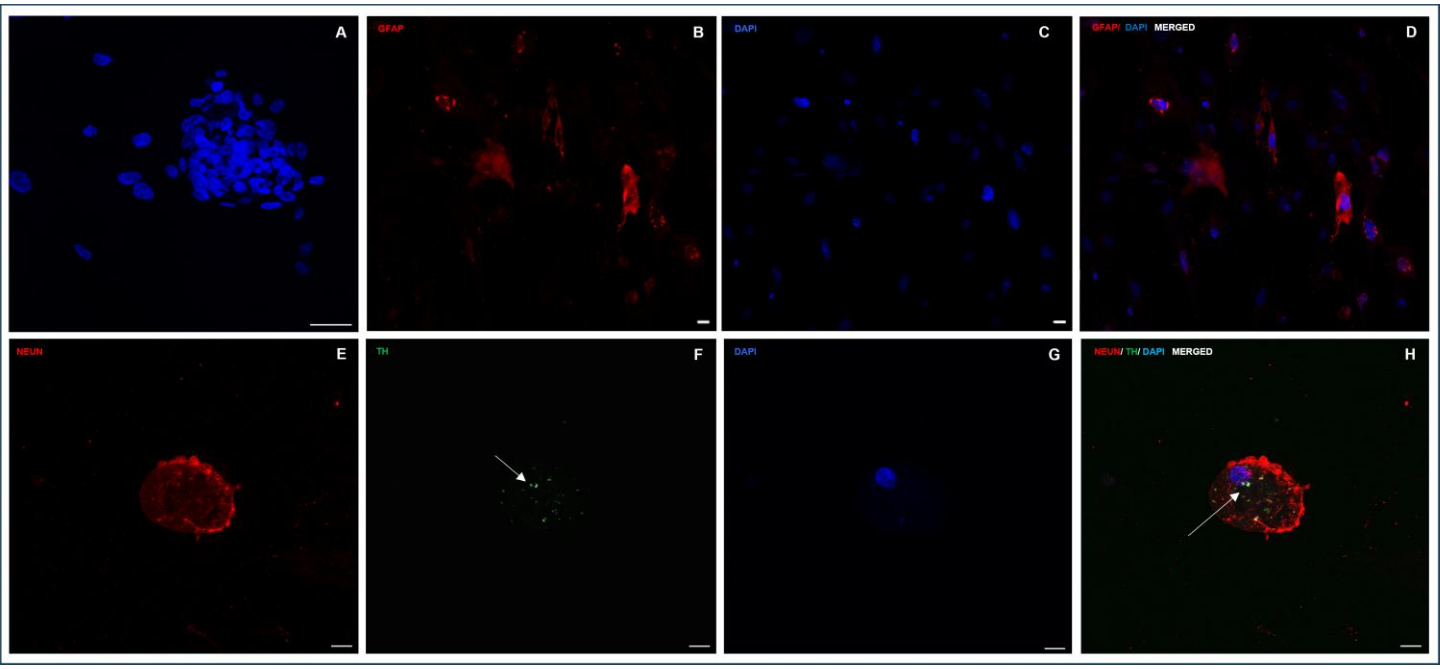

Figure S2.- Microphotographs showing cells from a negative control sample (A; blue staining, nuclei), single immunolocalization of the astrocyte marker GFAP (B; red staining), DAPI (C; blue staining, nuclei), and merged immunolocalization (D; red and blue); the neuron marker NeuN (E; red staining), DA neuron/precursor marker TH (F; green staining), DAPI (G; blue staining, nuclei), and merged immunolocalization of NeuN, TH and DAPI (D) and NeuN, TH and DAPI (H) in astrocytes and a dopaminergic neuron precursor growing in culture after thirty days of directed differentiation of OCC-NSPCs to the DA pathway. (B-D: HC, PL APO C52, 20X/0.75 magnification, dry; A, E-H: HC, PL APO CS2, 63X/1.40 magnification, oil). Nuclei were counterstained with DAPI. This figure supplements Figure 6 of the manuscript.

Figure S3: Individual and combined immunolocalization of NeuN, TH, and DAPI in NS generated during directed diferentiation of OCC-NSPC to the DA pathway.

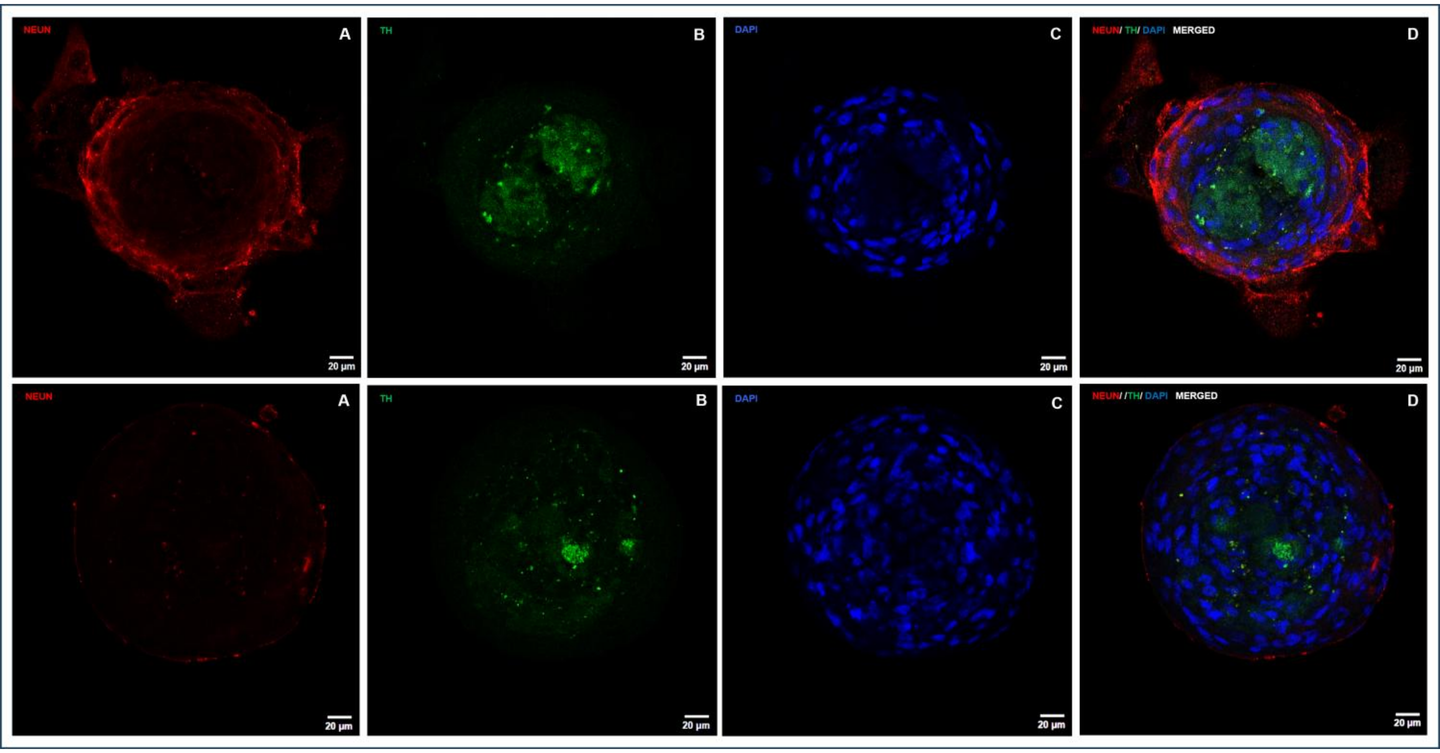

Figure S3.- Microphotographs showing single immunolocalization of NeuN (A; red staining), TH (B; green staining), DAPI (C; blue staining, nuclei), and merged immunolocalization of NeuN, TH and DAPI (D) in NS still generated in culture after thirty days of directed differentiation of OCC-NSPCs to the DA pathway. Images show medium sized NS with positive immunolocalization of NeuN in cells placed at the outer sheet (red; A, D) and TH (green; B, D) mainly immunolocalized in core cells and in cells placed at the outer sheet. (HC, PL APO C52, 20X/0.75 magnification, dry). Nuclei were counterstained with DAPI. This figure supplements Figure 6 of the manuscript.
